# Rhein modulates host purine metabolism in intestine through gut microbiota and ameliorates experimental colitis

**DOI:** 10.7150/thno.43528

**Published:** 2020-08-29

**Authors:** Jiawei Wu, Zhonghong Wei, Peng Cheng, Cheng Qian, Fangming Xu, Yu Yang, Aiyun Wang, Wenxing Chen, Zhiguang Sun, Yin Lu

**Affiliations:** 1 Jiangsu Key Laboratory for Pharmacolgy and Safety Evaluation of Chinese Materia Medica, School of Pharmacy, Nanjing University of Chinese Medicine, Nanjing 210023, PR China.; 2 State Key Laboratory Cultivation Base for TCM Quality and Efficacy, Nanjing University of Chinese Medicine, Nanjing University of Chinese Medicine, Nanjing 210023, PR China.; 3 Jiangsu Collaborative Innovation Center of Traditional Chinese Medicine (TCM) Prevention and Treatment of Tumor, Nanjing University of Chinese Medicine, Nanjing 210023, PR China.; 4 Jiangsu Provincial Second Chinese Medicine Hospital, The Second Affiliated Hospital of Nanjing University of Chinese Medicine, Nanjing 210017, China.

**Keywords:** Rhein, DSS-induced colitis, purine metabolism, uric acid, gut microbiota

## Abstract

**Background:** Gut microbiota, which plays a crucial role in inflammatory bowel diseases (IBD), might have therapeutic benefits for ulcerative colitis or Crohn's disease. Targeting gut microbiota represents a new treatment strategy for IBD patients. Rhein is one of the main components of rhubarb and exhibits poor oral bioavailability but still exerts anti-inflammatory effects in some diseases. Therefore, we investigated the effect of rhein on colitis and studied its possible mechanisms.

**Methods:** The chronic mouse colitis model was induced by four rounds of 2% dextran sulfate sodium (DSS) treatment. The mice were treated with 50 mg/kg and 100 mg/kg rhein daily, body weight, colon length, histological score, inflammatory cytokines in serum or intestine, and fecal lipocalin 2 concentration were determined. Th17 cell, Th1 cell and Th2 cell infiltration in the mesenteric lymph node were analyzed by flow cytometry. Metabolic profiles were collected by non-targeted metabolomics and key metabolic pathways were identified using MetaboAnalyst 4.0. We also assessed intestinal barrier permeability and performed 16s rDNA sequencing. *Lactobacillus sp.* was cultured, and fecal microbiota transplantation (FMT) was employed to evaluate the contribution of gut microbiota.

**Results:** Rhein could significantly alleviate DSS-induced chronic colitis. Uric acid was identified as a crucial modulator of colitis and rhein treatment led to decreased uric acid levels. We determined that rhein changed purine metabolism indirectly, while the probiotic *Lactobacillus* was involved in the regulation of host metabolism. Uric acid resulted in a worsened intestinal barrier, which could be rescued by rhein. We further confirmed that rhein-treated gut microbiota was sufficient to relieve DSS-induced colitis by FMT.

**Conclusion:** We showed that rhein could modulate gut microbiota, which indirectly changed purine metabolism in the intestine and subsequently alleviated colitis. Our study has identified a new approach to the clinical treatment of colitis.

## Introduction

Ulcerative colitis (UC) is an inflammatory bowel disease (IBD) restricted to the colonic mucosa and submucosa with characteristic ulcers or open sores 1. UC is normally characterized by diarrhea, bloody stools, abdominal pain and weight loss, and has a prevalence of 7.6-246 per 100000 individuals. The etiology of UC is still poorly understood, which is likely to involve immune dysfunction, genetic susceptibility and microbial imbalance 2. So far, there is no effective cure for ulcerative colitis or IBD, and the recurrence rate is high.

Rhein is the main component of several traditional Chinese medicines, including Rhubarb, Aloe, and Sennae Folium, and exerts multiple pharmacological effects, such as anti-inflammation, anti-tumor, anti-fibrosis and anti-oxidant 3-7. Rhein is believed to alleviate inflammation by inhibiting NF-κB and NLRP3 inflammasome activation in macrophages 3, and modulate the expression of Nrf2 and MAPK 8. It can also inhibit IKKβ to attenuate inflammation 9. Nevertheless, whether rhein could ameliorate colitis and its possible mechanisms remain poorly understood.

In past few years, accumulating evidences have supported that gut microbiota play an important role in the pathogenesis of UC 10. Normally, physical and biochemical barriers separate the microbiota from direct contact with intestinal epithelial cells and mucosa. Once the barriers are disrupted, the microbiota could successfully translocate from the lumen to the lamina propria and trigger the release of interleukin-1, interleukin-6 and interleukin-23, thus driving a pathogenic type 17 helper T-cell response, and finally causing immune cells recruitment 11. An imbalance among commensal, pathogens, and symbionts is believed to be involved in inflammatory bowel disease.

Numerous studies have confirmed that microbiota serves as the key player in IBD. Machiels et al. found that the abundance of butyrate-producing bacteria such as *Roseburia hominis* and *Faecalibacterium prausnitzii* are decreased in patients with Crohn's disease 12. Michielan and colleagues reported that patients with IBD suffered depletion of mucus, and exhibited decreased expression of tight junction proteins and microbiota translocation 13. Restore the microbiota homeostasis and intestinal barrier function may be an efficient way to counter UC or IBD. Some specific bacterial strains, identified as the probiotics, have been shown to modulate IBD directly. For example, Wang reported that *Lactobacillus acidophilus* and *Clostridium butyricum* could ameliorate colitis by strengthening the gut barrier function and decreasing inflammatory factors 14. VSL#3, a probiotic mixture, can also alleviate colitis in mouse models 15, 16, suggesting a promising treatment for patients. However, whether rhein could modulate microbiota remains unknown.

Here we report that rhein alleviated DSS-induced colitis through modulating the composition of gut microbiota and restored intestinal barrier function. Oral gavage of rhein resulted in decreased inflammation level and modified metabolic profile. We found altered purine metabolism and decreased uric acid levels in the intestine in the rhein-treated group by *Lactobacillus*. Rhein caused a significant increase in the *Lactobacillus* level, one of the major probiotics, and decreased intestinal permeability. Collectively, our study demonstrated the anti-inflammatory effects of rhein in a DSS-induced chronic colitis model mainly through regulating the microbiota.

## Results

### Rhein alleviated DSS-induced chronic colitis in mice

**Figure 1:**
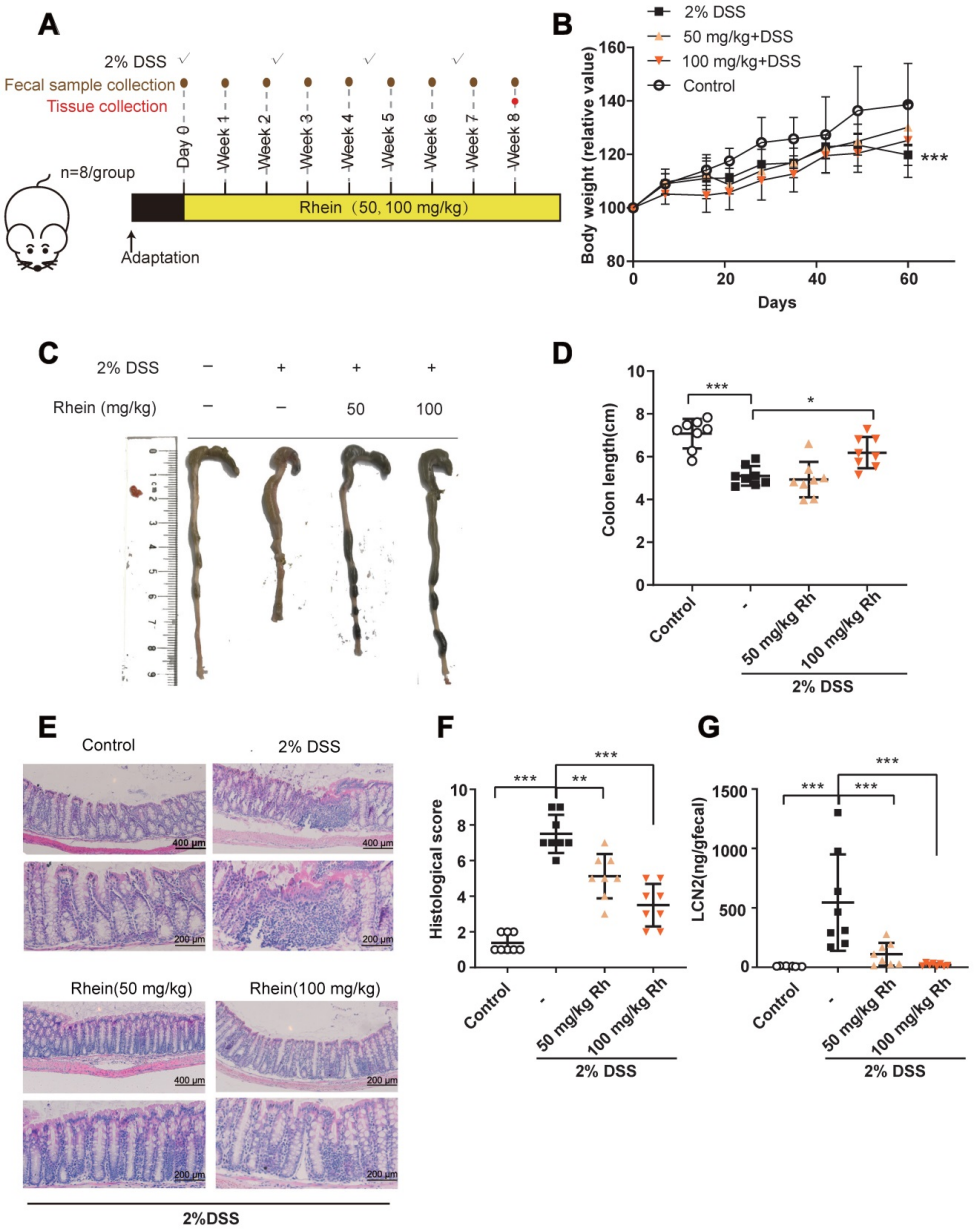
** Rhein alleviated DSS-induced chronic colitis.** (**A**) Experiment design. (**B**) Relative bodyweights during mouse model development (n = 8). (**C**) Representative colon pictures from each group. (**D**) Colon length in each group (n = 8). (**E**) Representative H&E staining of colon tissue sections from each group. Scale bar, above: 400 µm, below: 200 µm. (**F**) Histological score in each group (n = 5). (**G**) Fecal level of the inflammatory marker LCN2 in each group (n = 8). *P < 0.05, **P < 0.01, ***P < 0.001 versus DSS group. DSS: dextran sulfate sodium; Rh: rhein. At least two independent experiments were performed.

We evaluated whether rhein could exert anti-inflammatory effects in a mouse chronic colitis model **(Figure 1A)** 17. After 4 rounds of 2% DSS treatment, mice suffered severe colonic inflammation. Treatment with 100 mg/kg rhein alleviated colon shortening while both 50 mg/kg and 100 mg/kg rhein-treated groups exhibited less immune cell infiltration and tissue damage than the DSS Group **(Figure 1C-F)**. Chronic DSS treatment caused a reduction in body weight, which only showed a slight increase following rhein treatment **(Figure 1B)**, possibly due to the fat-reducing effect of rhein as previously reported 18. DSS treatment caused a marked increase in fecal lipocalin 2 (LCN2), a sensitive marker of intestinal inflammation in mice 19, which could be reversed by 50 mg/kg and 100 mg/kg rhein treatment **(Figure 1G)**.

**Figure 2:**
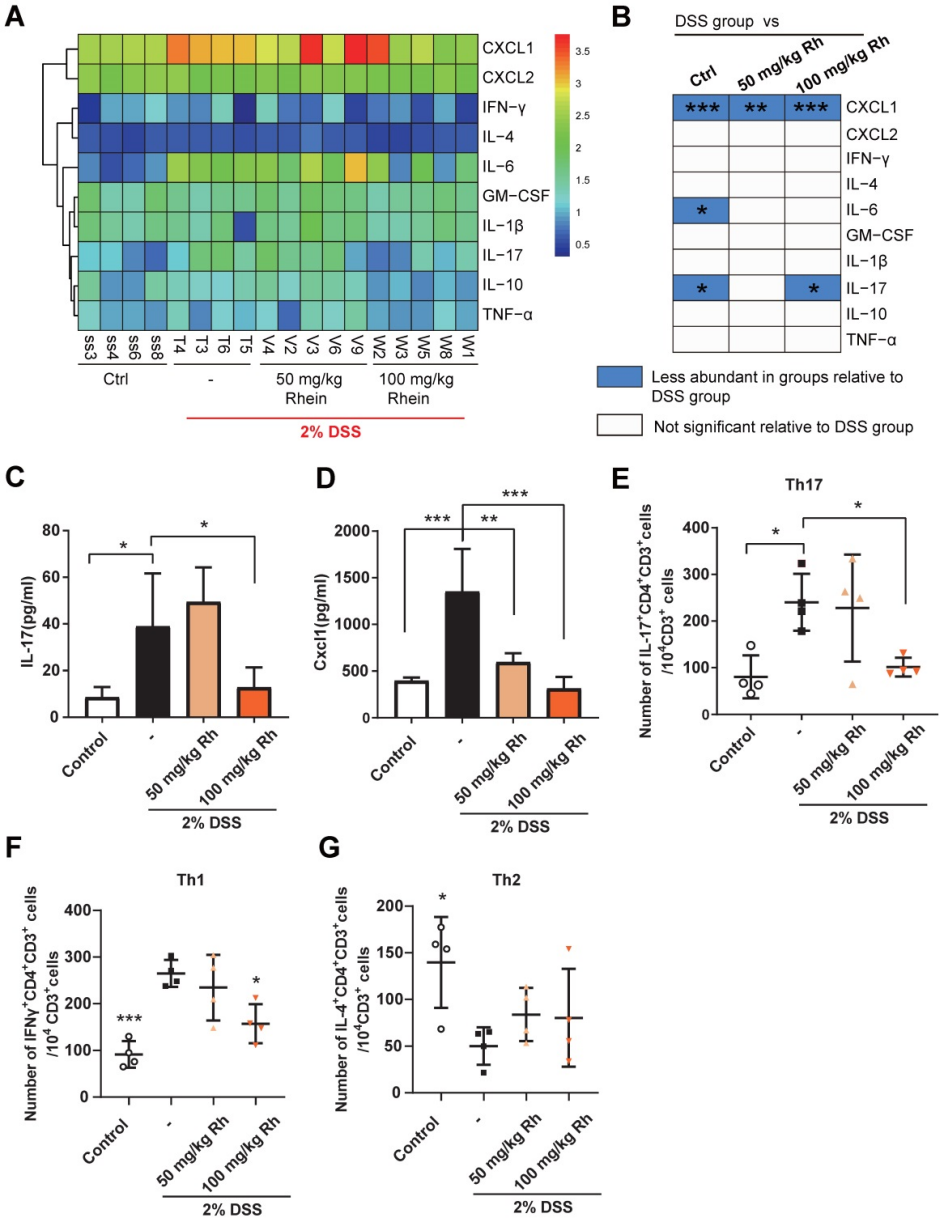
** Rhein decreased inflammatory cytokines production during DSS-induced colitis.** (**A**) Heatmap of ten specific cytokine levels in serum, including CXCL1, CXCL2, IFN-γ, IL-4, IL-6, GM-CSF, IL-1β, IL-17, IL-10, and TNF-α (n = 4-5). (**B**) Cytokines and chemokines from panel A and comparisons of DSS and other groups. Blue entries indicate cytokines/chemokines that were less abundant in various groups compared to the DSS group. (**C**) SerumIL-17 concentration in each group (n = 4-5). (**D**) Serum Cxcl1 concentration in each group (n = 4-5). (**E**) Flow cytometry analysis of Th17 cells (CD3^+^CD4^+^IL-17A^+^) per 10^4^ CD3^+^ cells in each group (n = 4). (**F**) Flow cytometry analysis of Th1 cells (CD3^+^CD4^+^IFNγ^+^) per 10^4^ CD3^+^ cells in each group (n = 4). (**G**) Flow cytometry analysis of Th2 cells (CD3^+^CD4^+^IL-4^+^) per 10^4^ CD3^+^ cells in each group (n = 4). For the gating strategy see **Figure S1D.** *P < 0.05, **P < 0.01, ***P < 0.001, compared with DSS group. DSS: dextran sulfate sodium; Rh: rhein; Ctrl: control. At least two independent experiments were performed.

Colitis can also result in increased production of pro-inflammatory cytokines or chemokines. We employed the Luminex strategy to quantify a panel of cytokines and chemokines. DSS chronic treatment caused a marked increase in IL-17A and CXCL1 production in mouse serum** (Figure 2A-D)**. Notably, IL-10 production was not affected. The significantly high colonic concentration of IL-17, CXCL1, and IFN-γ in DSS group mice could be reversed by 100 mg/kg rhein treatment, while IL-10 concentration displayed no significant differences among groups (**Figure S1A**). Also, serum IL-17/IL-10 ratio was upregulated after DSS treatment, while the colonic IL-17/IL-10 ratio was significantly downregulated by a high dose of rhein (**Figure S1B-C**). Since IL-17 is mainly secreted by Th17 cells 20, closely related to colonic inflammation 21, we next examined Th17 cells in mouse mesenteric lymph nodes (MLN). Consistent with the IL-17 level in serum, mice treated with 100 mg/kg rhein showed fewer Th17 cells in MLN, while 50 mg/kg rhein treatment had no effect **(Figure 2E, Figure S1D)**. Th1/Th2 cells imbalance is also an important event in colitis. As is shown in **Figure 2F-G**, Th1 cells were dramatically expanded during colitis while Th2 cells were decreased in DSS group. Interestingly, rhein treatment only decreased Th1 cells while showed barely no effect on Th2 cells. Rhein treatment without DSS did not lead to any noticeable side effects or pro-inflammatory responses **(Figure S2)**. These data suggested that rhein treatment ameliorated DSS-induced colitis, and 100 mg/kg rhein treatment was more effective than 50 mg/kg rhein treatment.

### Rhein altered purine metabolism and decreased uric acid level

**Figure 3:**
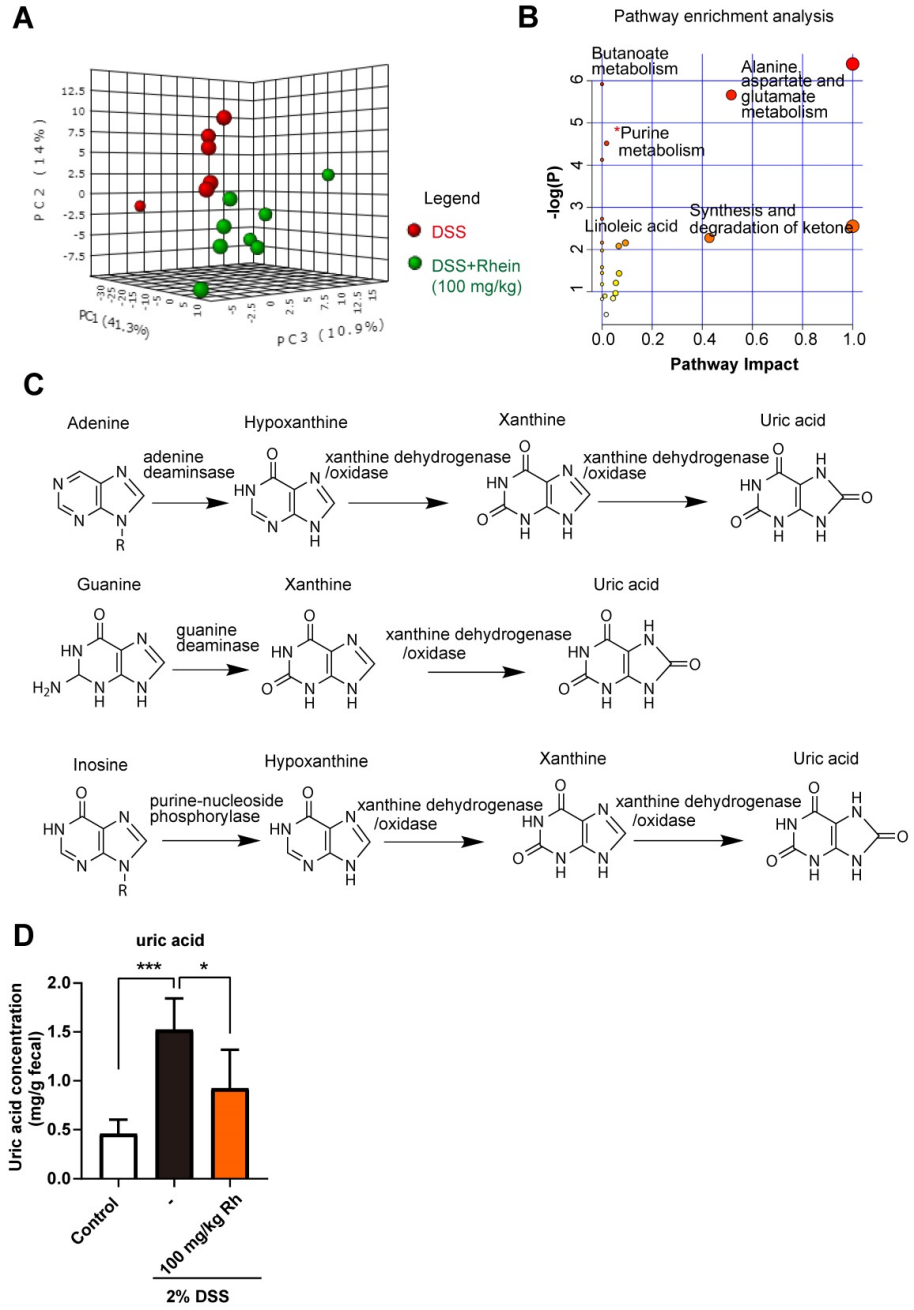
** Rhein shifted metabolic profiles in the mouse intestine.** (**A**) 3D-PCA analysis on metabolites in feces revealed distinct metabolite composition between DSS and rhein-treated groups (n = 6-8). (**B**) Pathway enrichment analysis of differentially expressed metabolites, see also Figure S2. (**C**) Schematic diagram of purine metabolism. Uric acid is the final metabolite. (**D**) Relative uric acid concentration in each group normalized by peak intensity over the stool weight of paired samples (n = 6-8). PCA: principal component analysis. DSS: dextran sulfate sodium. *P < 0.05, compared with the DSS group.

Metabolic changes are considered as one of the most important hallmarks of intestinal bowel disease 22. During inflammation, the intestinal metabolic profile shifts and host metabolic pathways may be altered. Based on this notion, we used non-targeted metabolomics to identify key metabolites and metabolic pathways that might be altered in the mouse intestine. A total of 159 metabolites were identified in feces, and 3D-PCA analysis revealed differences between DSS and 100 mg/kg rhein groups **(Figure 3A)**. Next, we chose differentially expressed metabolites (fold change>2, *p*-value <0.05) for further analysis and identified 23 differentially expressed metabolites between the two groups **(For detailed metabolite information see Figure S3A)**.

To further investigate the possible metabolic pathways, we performed pathway enrichment analysis using Metaboanalyst 4.0 (https://www.metaboanalyst.ca/). Among the potential pathways, the purine metabolism pathway attracted our attention** (Figure 3B, Figure S3B)**. Purines perform many important functions in cells, the formation of the monomeric precursors of nucleic acids DNA and RNA being the most relevant one **(Figure 3C)**. Under normal conditions, the enzymes involved in the purine metabolism maintain a balanced ratio between synthesis and degradation in cells. Once the balance is disrupted, it can lead to excessive uric acid production, the final metabolite in purine metabolism 23. Thus, we analyzed uric acid levels in feces and found that DSS caused a marked increase in uric acid concentration, while rhein treatment abrogated elevated uric acid concentration **(Figure 3D)**. Taken together, we confirmed purine metabolism pathway was altered during colitis, rhein treatment resulted in normalized purine metabolism and lowered uric acid levels in intestine.

### Elevated uric acid directly led to intestinal barrier damage

**Figure 4:**
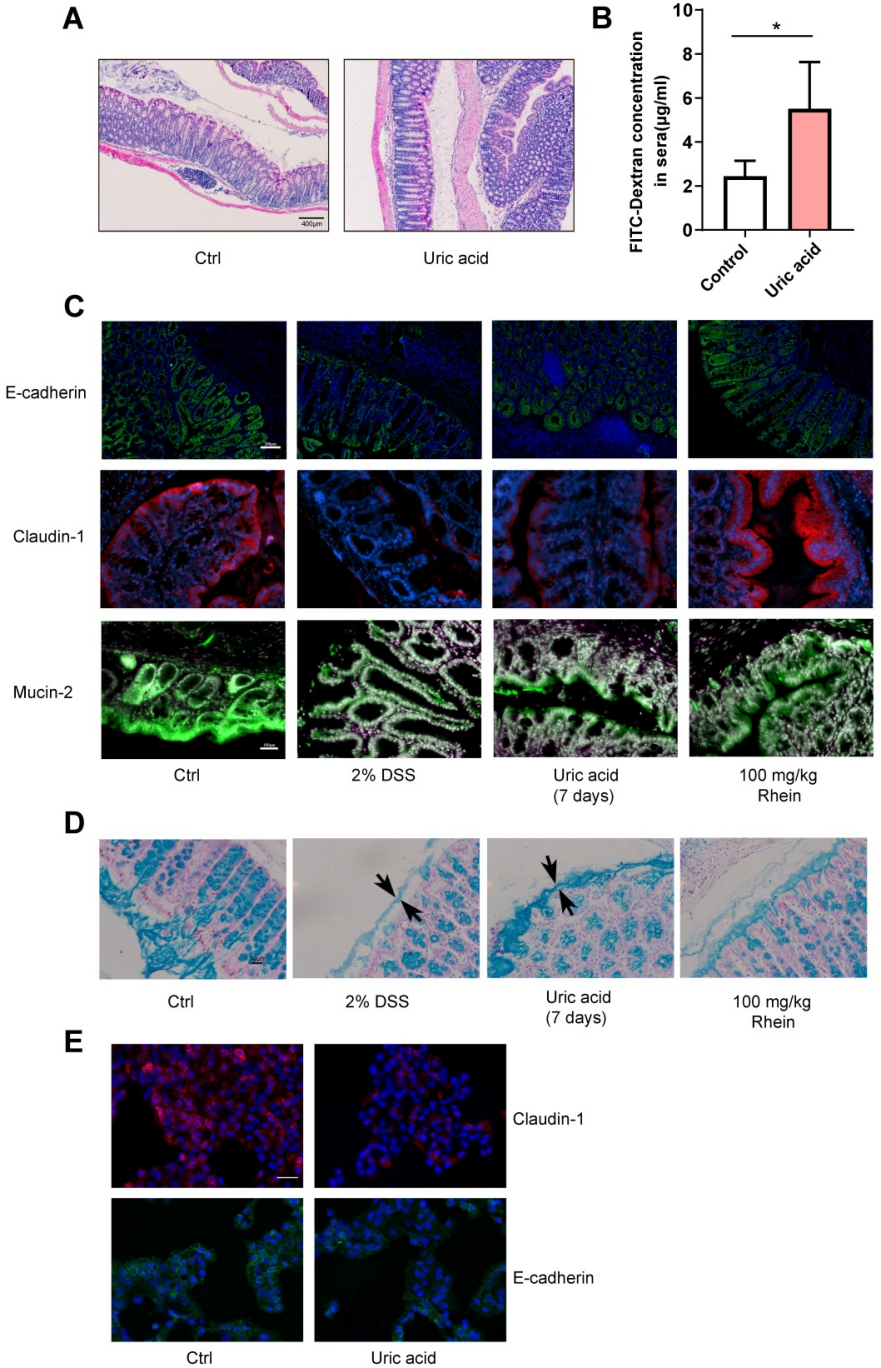
** Uric acid disrupted intestinal integrity in the absence of DSS.** Uric acid 1g/kg was orally gavaged for 7 days. (**A**) Representative H&E staining of colon tissue sections from each group. Scale bar, 400 µm. (**B**) Intestinal leakage measured by FITC-Dextran concentration in serum (n = 4). **C.** Immunofluorescence analysis on E-cadherin, claudin-1 and mucin-2 in colon sections from different groups. Representative images are shown. Scale bar, 200 µm. (**D**) Alcian Blue staining on colon tissue sections; the black arrow shows the thickness of the inner mucus layer. Representative images are shown. Scale bar, 50 µm. (**E**) NCM-460 cells treated with uric acid for 24 h and representative immunofluorescence images of claudin-1 and E-cadherin are shown. Scale bar, 200 µm. *P < 0.05, compared with the control group. Three independent experiments were performed.

The elevated uric acid concentration in serum or urine is strongly correlated to several disorders, including gout, kidney and vascular diseases 24. During IBD, uric acid is also elevated and may contribute to the development of uric acid nephrolithiasis 25. Recent studies have shown that uric acid plus DSS treatment could result in worsened intestinal epithelial damage and colitis compared to DSS treatment alone 26. Therefore, we tested whether uric acid alone could lead to the disruption of intestinal barrier integrity. Uric acid administration alone for 7 days did not cause obvious inflammation with infiltration from a few immune cells **(Figure 4A)**. However, we observed greater intestinal permeability measured by leakage of FITC-dextran into the blood **(Figure 4B)**. The expression of tight junction protein claudin-1 and cell adhesion protein E-cadherin were decreased, as shown in **Figure 4C**. It has been reported that the colonic mucus layer is the key component forming the intestinal barrier and is critical for gut homeostasis 27. We also observed thinner mucus layer and less mucin-2 secreted by goblet cells **(Figure 4C-D)**. In contrast, rhein restored claudin-1, E-cadherin expression, and mucus secretion. We further tested this effect *in vitro* using human normal colon NCM-460 cells. Consistent with our findings *in vivo*, uric acid treatment reduced expression of claudin-1 and E-cadherin **(Figure 4E)**. Notably, uric acid treatment plus 100 mg/kg rhein treatment also exhibited strong protective effects against DSS-induced chronic colitis (**Figure S4**), indicating the protective effects of rhein were not influenced by the supplementation of uric acid. These data demonstrated that rhein treatment could abolish the enhanced intestinal permeability caused by uric acid.

### Rhein did not lower uric acid concentration directly

We next explored the underlying mechanisms by which rhein treatment altered purine metabolism. Since uric acid forms in the final step of purine metabolism pathway as a product of xanthine and hypoxanthine by xanthine dehydrogenase/oxidase (XDH), we first examined whether rhein could affect XDH at the transcriptional level. We treated NCM-460 cells with various rhein concentrations and did not find any difference between the two groups **(Figure S5A)**. Next, we determined uric acid concentration in the supernatant of cultured cells treated with rhein, and did not observe any significant changes **(Figure S5B)**. These data indicate that rhein may not be the direct modulator of purine metabolism in the intestine.

### Rhein treatment altered gut microbiota composition and increased *Lactobacillus* level leading to decreased uric acid levels

**Figure 5:**
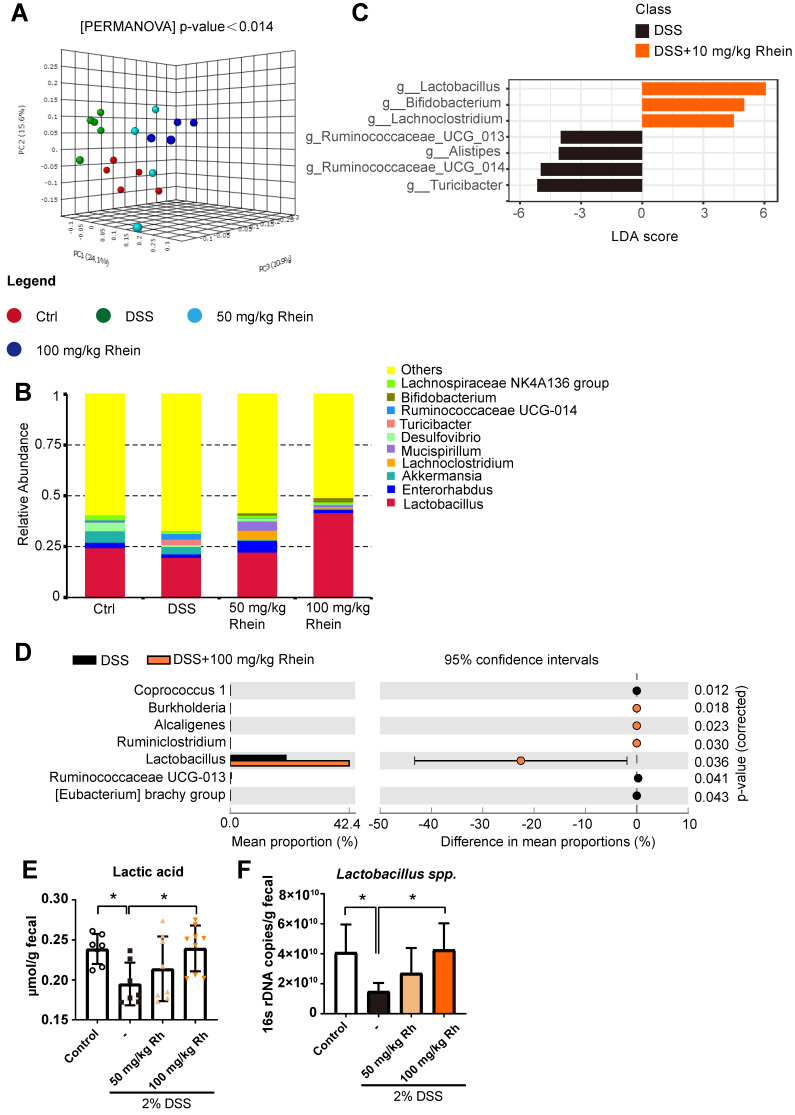
** 16s rDNA sequencing revealed altered microbiota composition after rhein treatment.** (**A**) UniFrac-based 3D-PCoA analysis with the PERMANOVA significant test for each group (n = 5). (**B**) Relative abundance of microbial taxa was determined at the genus level. Top 10 abundances are shown. (**C**) Linear discriminant analysis (LDA) scores derived from LEfSe analysis, showing biomarker taxa at the genus level (LDA score) of >4 and a significance of *P* <0.05 determined by the Wilcoxon signed-rank test. (**D**) STAMP analysis uncovered the differences between DSS and rhein-treated groups. Genera with a significance of *P* < 0.05 are shown. (**E**) 16s rDNA copies of *Lactobacillus spp.* measured by quantitative PCR. (**F**) Fecal lactic acid concentration in each group (n = 7-8). *P < 0.05, compared with the DSS group. DSS: dextran sulfate sodium; Rh: rhein; Ctrl: control.

Although rhein treatment did not directly reduce uric acid concentration, it resulted in lower uric acid levels *in vivo*. We sought to explain this paradoxical phenomenon. Intestinal lumen is a habitat for numerous microbes including bacteria, and host-microbiota interaction may affect disease states 28. Unlike humans, bacteria possess the ability to degrade uric acid by uric acid oxidase (uricase), and specific bacterial strains also have XDH inhibitory activities 29. We hypothesized that bacteria might be the main cause of reduced uric acid in mouse intestine. To test this hypothesis, we utilized 16s ribosomal RNA gene sequencing to determine whether rhein treatment could alter gut microbiota composition. The UniFrac-based 3D-principal coordinate analysis revealed distinct clustering of microbiota composition for each group **(Figure 5A-B)**. To identify the different bacteria between DSS and 100 mg/kg rhein groups, we performed LEfSe analysis and selected genera based on the LDA score >4. Notably, we observed an overrepresentation of distinct bacterial genus *Lactobacillus* in 100 mg/kg rhein-treated group compared to the DSS group **(Figure 5C)**. We further confirmed this finding using STAMP analysis and quantitative PCR **(Figure 5D-F)**. All analyses showed a significant abundance of *Lactobacillus* after rhein treatment. Consequently, lactic acid concentration in feces was increased **(Figure 5E)**.

**Figure 6:**
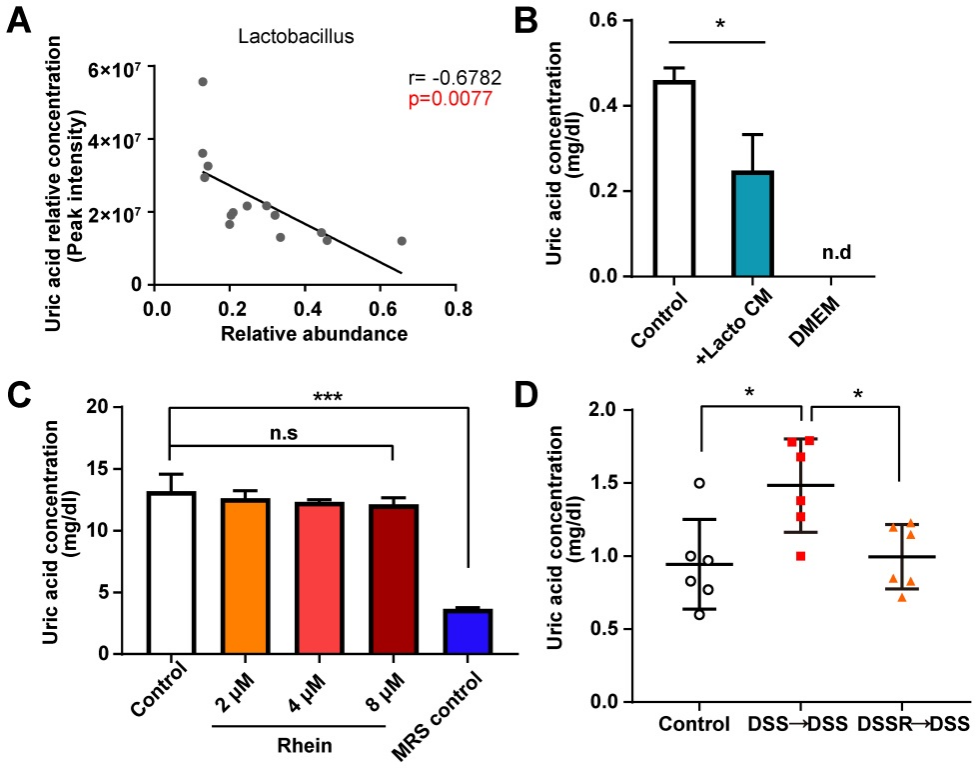
** Uric acid lowering effects of *Lactobacillus*.** (**A**) Correlation analysis between uric acid level and paired* Lactobacillus* abundance in feces. (**B**) Uric acid concentration of NCM-460 cells treated with fermentation products derived from *Lactobacillus sp.* was determined (n = 3). (**C**) *Lactobacillus sp.* treated with different doses of rhein and uric acid levels in culture media was detected (n = 3). (**D**) Uric acid concentration in each group (n = 6). For B-C, *P < 0.05, ***P < 0.001, compared with the control group. For D, *P < 0.05, **P < 0.01, compared with the DSS group. Lacto CM: *Lactobacillus sp.* culture media; DSS: dextran sulfate sodium; FMT: fecal microbiota transplantation; n.s: not significant. Three independent experiments were performed.

*Lactobacillus* is one of the most well-known probiotics, the supplementation of which could alleviate IBD 30-32. However, the underlying mechanisms remain unclear. Based on the evidence above, we next determined the relationship between *Lactobacillus* level and paired uric acid concentration using correlation analysis **(Figure S6)**. As expected, *Lactobacillus* abundance negatively correlated with uric acid concentration in mouse intestine, suggesting the pivotal role of *Lactobacillus* in purine metabolism **(Figure 6A)**. Other differential bacterial genera, such as *Alcaligenes* and *Ruminiclostridium,* did not appear to be involved in purine metabolism **(Figure S6)**. Recently, *Lactobacillus gasseri* was shown to decrease purine levels in the intestine, and fermentation products of *Lactobacillus* had urate-lowering effects 33. To confirm this effect, we cultured *Lactobacillus sp. in vitro* and co-cultured the supernatant with NCM-460 cells. After 24 h, uric acid concentration was measured. We found that *Lactobacillus sp.* supernatant decreased uric acid concentration, suggesting the uric acid lowering effect of *Lactobacillus* fermentation products **(Figure 6B)**.

**Figure 7:**
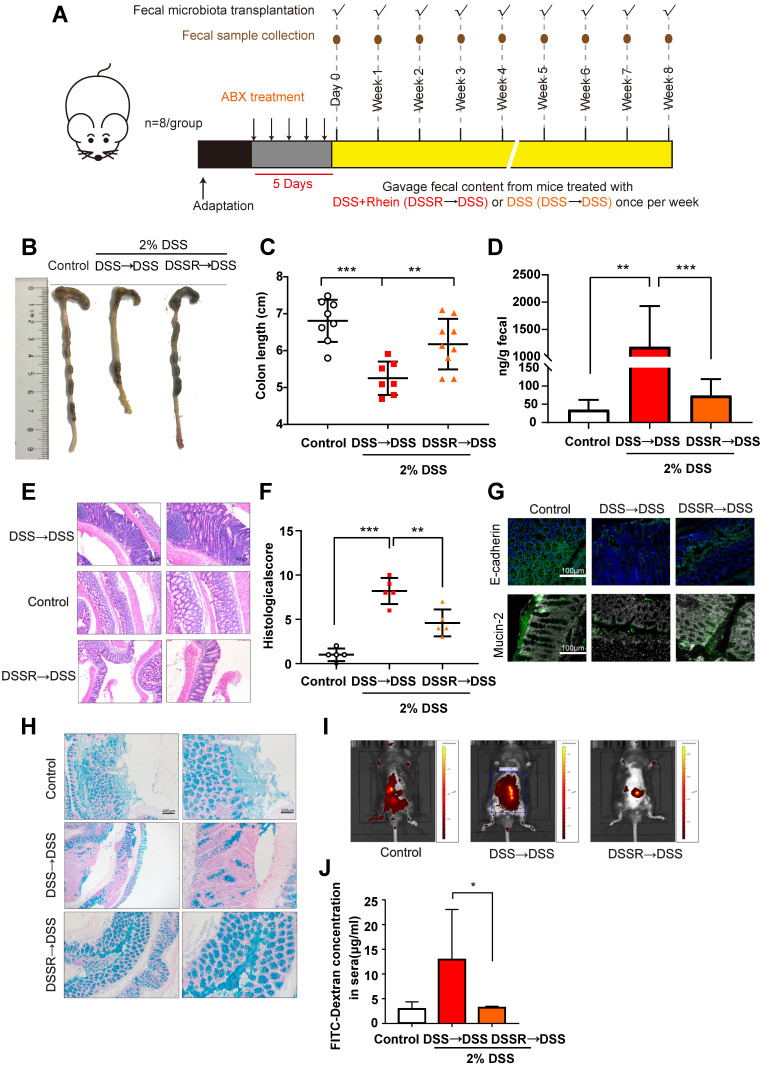
** FMT alleviated DSS-induced colitis.** (**A**) Schematic diagram of the FMT procedure. (**B**) Representative colon pictures from each group. (**C**) Colon length in each group (n = 8). (**D**) Fecal level of the inflammatory marker LCN2 in each group (n = 8). (**E**) Representative H&E staining of colon tissue sections from each group. Scale bar, left panel: 400 µm; right panel: 200 µm. (**F**) Histological score in each group (n = 5). (**G**) Immunofluorescence analysis of E-cadherin and mucin-2 in colon sections from different groups. Representative images are shown. Scale bar, 100 µm. (**H**) Alcian Blue analysis of each group, Representative images are shown. Scale bar, left panel: 400 µm; right panel: 200 µm. (**I**) *In vivo* imaging of FITC-Dextran. (**J**) Intestinal leakage measured by FITC-Dextran concentration in serum (n = 3). DSS→DSS group: mice were transplanted with microbiota from the previous DSS group followed by the induction of colitis. DSSR→DSS group: mice were transplanted with microbiota from the previous 100 mg/kg rhein+DSS group followed by the induction of colitis.*P < 0.05, **P < 0.01, ***P < 0.001 versus DSS→DSS group. At least two independent experiments were performed.

We also treated *Lactobacillus sp.* with rhein and detected uric acid levels. Rhein treatment did not change uric acid production in *Lactobacillus sp.*, suggesting that the change in the uric acid level might have originated from intestinal epithelial cells **(Figure 6C)**. To gain a better understanding of the gene expression of key enzymes involved in purine degradation, we predicted bacterial metagenome content from 16s rDNA gene-based microbial compositions using the PICRUSt2 algorithm. Expression of key enzymes in purine metabolism, including *xdh, ade, xdhA, xdhB, yagT* and *guaB* was not upregulated in the DSS group compared to 100 mg/kg group (**Figure S7**). In fact, we observed a slight increase of these enzymes in the rhein-treated group, ruling out the possibility that difference in uric acid concentration was mainly caused by bacteria. Finally, we conducted fecal microbiota transplantation (FMT) to verify whether microbiota or rhein caused the uric acid lowering effects. Fecal microbiota from DSS- or 100 mg/kg rhein-treated mice were transplanted into DSS recipients (**Figure 7A**). As expected, FMT led to reduced uric acid in the DSS-induced mouse colitis models **(Figure 6D)**. These results showed that rhein could alter gut microbiota composition and lead to increased *Lactobacillus* abundance, reducing uric acid production in intestinal epithelial cells.

### FMT ameliorated DSS-induced colitis and restored intestinal barrier function

Next, we used FMT to determine whether gut microbiota altered by rhein had therapeutic benefits for colitis. Fecal microbiota from DSS- or 100 mg/kg rhein-treated mice were transplanted into DSS recipients. FMT was verified by 16s rDNA sequencing and qPCR (**Figure S8A-B**). We further analyzed the 16s rDNA data obtained from donor mice and recipient mice. STAMP analysis showed different bacteria strains between DSS+Rhein and FMT group, including *Synechococcus*, *Prochlorococcu*, *Oscillibacter*, *Candidatus Soleaferrea*, *Candidatus Actinomarina* and *OM60 (NOR5) clade* (**Figure S8C**). However, the relative abundance of these bacteria are extremely low (<0.01%), thus it is unlikely to exert biological functions in different groups. Linear Discriminant Analysis (LDA) Effect Size (LEfSe) did not identified any significant features under the criteria: Log LDA score>2, *P*-value cutoff <0.05. These data further supporting the successful transference of microbiota from donor mice to recipient mice. As displayed in **Figure 7A-E**, FMT significantly alleviated DSS-induced colitis and decreased immune cell infiltration in colon tissue sections. Furthermore, the expression of E-cadherin and claudin-1 were increased in the FMT group, suggesting mucus layer thickness was restored **(Figure 7F-G)**. Importantly, we confirmed that FMT protected gut leakage using *in vivo* imaging of FITC-dextran leakage **(Figure 7H-I)**. The FMT group also showed upregulation of *Lactobacillus* levels compared to the DSS group (**Figure S8D**). These data demonstrated FMT ameliorated DSS-induced colitis and prevented intestinal barrier permeability; further supporting the notion that microbiota changed by rhein during DSS-treatment is beneficial to colitis.

## Discussion

The last few decades have witnessed the rapid development of next-generation sequencing technologies, which allow us to unravel the microbiota residing in the intestine, skin, oral cavity and lungs. It is known that microbiota participates in host diseases such as obesity and liver, brain, cardiovascular, and inflammatory bowel diseases 34. Changes in gut microbiota composition have been repeatedly reported in IBD patients. Besides, dysbiosis, usually linked to reduced bacterial diversity and the imbalance between each bacterial phyla, occurred in IBD patients 35. Dysbiosis often leads to the decrease of short-chain fatty acids in the intestine, which are the important energy source of intestinal epithelial cells, and may cause increased intestinal permeability and initiate inflammation 36. Based on this notion, utilizing probiotics or fecal microbiota transplantation to restore the gut homeostasis may be an efficient strategy to treat IBD 37.

Here we demonstrated that chronic rhein treatment alleviated DSS-induced colitis. We employed a metabolomics strategy to search for the possibly altered metabolites and metabolic pathways and identified modifications in the purine metabolism pathway. The altered purine metabolism resulted in increased uric acid concentration. *In vitro* experiments showed rhein did not affect uric acid production and *XDH* expression, suggesting rhein did not directly regulate uric acid production. The detailed mechanisms of dextran sodium sulfate remain unclear but are likely due to the altered bacteria composition and disrupted intestinal barrier. Thus, bacteria could successfully translocate from the intestinal lumen into the lamina propria and trigger an inflammatory response 38. The high uric acid level in the intestine could directly increase intestinal barrier permeability; however, uric acid alone did not cause inflammatory responses, indicating other key regulators may be involved in DSS-induced colitis.

Chinese medicine is attracting increasing attention worldwide due to its powerful therapeutic effects and few side effects. However, there are still concerns about how the poorly absorbed compounds in Chinese medicine exert therapeutic effects. Numerous studies have indicated a close relationship between Chinese medicine and gut microbiota. The low absorption provides an opportunity for direct contact between the compounds and microbes. Such an interaction between gut microbiota and active compounds include: (a) direct modulation of gut microbiota composition by compounds in Chinese medicine, (b) active compounds regulate the metabolism of gut microbiota, and (3) specific bacterial strains transform active compounds 39. Thus, gut microbiota may be the key to explaining how low oral bioavailability compounds still exert strong pharmacological effects *in vivo*.

Based on this notion, we found that rhein did not alter uric acid production directly since *in vitro* rhein treatment did not cause a noticeable change in uric acid concentration or *XDH* expression. However, we found a significant increase in the *Lactobacillus* level in mouse intestine after rhein treatment. Interestingly, *Lactobacillus* level negatively correlated with uric acid level, indicating the uric acid lowering effect of *Lactobacillus*. Indeed, fermentation products of *Lactobacillus sp.* have been shown to lower uric acid and alleviate hyperuricemia. Moreover, *Lactobacillus gasseri* could directly utilize purine and decrease the absorption of food-derived purines in the human intestine 40. As anticipated, upon culturing NCM-460 cells with the fermentation product of *Lactobacillus sp.* we found decreased uric acid level, confirming the uric acid lowering effect of *Lactobacillus*. However, how rhein altered microbiota composition and the detailed mechanisms underlying the uric acid lowering effect of *Lactobacillus sp.* remain unclear and need further investigation.

Finally, we used FMT to verify the pharmacological effects of rhein in the DSS model. FMT relieved colonic inflammation and decreased intestinal permeability. We also observed lower uric acid concentration in the FMT group, demonstrating the modulation of uric acid by gut microbiota. In conclusion, our study provided evidence for the anti-inflammatory effects of rhein in a mouse model of colitis and its regulation of intestinal microbiota. Hence, Rhein treatment or targeting specific microbiota such as *Lactobacillus* offers a new strategy for the treatment of ulcerative colitis or IBD.

## Materials and Methods

### Animals

Male C57BL/6J nju mice aged 6 weeks were purchased from Nanjing Biomedical Research Institute of Nanjing University (Nanjing, China). Mice were housed under standard laboratory conditions (room temperature: (22 ± 2) ℃; humidity: (50 ± 5) %) with a light/dark cycle of 12/12 h (lighting on at 7:00 a.m.). All experimental protocols were approved by the Animal Care and Use Committee of Nanjing University of Chinese Medicine (Nanjing, China) and conducted conforming to the Guidelines for the Care and Use of Laboratory Animals (ACU170904).

### Induction of chronic colitis with DSS

All mice were divided into four groups: control group, DSS model group, DSS plus low dose rhein Goup (50 mg/kg) and DSS plus high dose rhein (100 mg/kg). Colitis was induced in mice as previously reported with slightly modifications 38. Briefly, 6-week-old mice were fed with 2% DSS (MP Biomedicals, Canada) in drinking water for 5 days and replaced by autoclaved water for another 10 days. Body weight was measured weekly. After 4 rounds of treatment, mice were sacrificed on day 60 (approximately 15 weeks old) or reached humane end points, and the length of colon were measured, opened longitudinally and intestinal contents were collected and used for further investigation. 50 mg/kg or 100 mg/kg Rhein (Nanjing Jingzhu Bio-technology CO., LTD., Nanjing, China) was administrated by oral gavage since the induction of colitis until the end of experiment. For uric acid treatment, 1 g/kg uric acid (Sigma) was administrated by oral gavage daily for 7 days.

### Haematoxylin and eosin staining and histopathologic analysis

Whole colon tissues were randomly selected (n = 5 per group), opened longitudinally, fecal contents were collected and tissues were washed with PBS. Colons were “Swiss rolled” and fixed with Carnoy's fixative (dry methanol: chloroform: glacial acetic acid in the ratio 6:3:1). The colons were fixed overnight, washed in methanol 2×30 min, ethanol 2×15 min, ethanol/xylene (1:1) 15 min and xylene 2×15 min, followed by embedding in paraffin. Tissues were sectioned at 5 μm thickness and stained with haematoxylin and eosin (H&E) using standard protocols. Histological scores were assessed as previously described based on the degree of epithelial damage and inflammatory infiltrate in the mucosa, submucosa and muscularis/serosa 41. The four individual scores per colon were added, resulting in a total scoring range of 0-12 per mouse. Pathobiological examination were conducted and scored in a blinded manner.

### Quantification of fecal LCN2 by ELISA

Mouse fecal samples were collected at the end of experiment and preserved at -80 °C. Frozen fecal samples were reconstituted in PBS containing 0.5% Tween 20 to a final concentration of 100 mg/ml and vortexed for 20 min to produce a homogenous fecal suspension. These samples were then centrifuged for 10 min at 14,000 g and 4 °C. LCN2 levels were estimated in the supernatants using Mouse Lipocalin-2/NGAL DuoSet ELISA kit (R&D systems, Shanghai) according to the manufacturer's instructions. Final results were normalized by fecal weight.

### Measurement of cytokines in serum

The absolute concentration of cytokines in serum were quantified by MILLIPLEX MAP Mouse Cytokine/Chemokine Magnetic Bead Panel (MCYTOMAG-70K-10, Millipore) containing CXCL1, CXCL2, IFN-γ, IL-4, IL-6, GM-CSF, IL-1β, IL-17, IL-10 and TNF-α according to the manufacturer's instructions.

### Flow cytometry analysis

Mesenteric lymph nodes were collected, ground and filtered through 100 μm cell strainers. Cells were counted and stimulated using Cell stimulation Cocktail plus transport inhibitors 500× (eBioscience) for 4 h. At the end of stimulation, cells were harvested, fixed using IC fixation buffer and permeabilized using 1× permeabilization buffer (eBioscience). Cells were divided into two parts then stained with antibodies as follows: (a) anti-CD3-APC (17-0032-82, eBioscience), anti-CD4-FITC (MA5-17443, eBioscience), and IL-17A-PerCP-Cyanine5.5 (45-7177-82, eBioscience); (b) anti-CD3-APC (17-0032-82, eBioscience), anti-CD4-FITC (MA5-17443, eBioscience), anti-IL-4-PE (12-7041-82, eBioscience) and anti-IFN gama-PerCP-Cyanine5.5 (45-7311-82, eBioscience). Single cell suspensions were examined using BD Accuri C6 and the data were analyzed using C6 software. The number of IL-17A^+^CD4^+^CD3^+^, IFNγ^+^ CD4^+^CD3^+^ and IL-4^+^ CD4^+^CD3^+^cells were calculated.

### Metabolomics analysis

For fecal metabolomics, approximate 50 mg fecal content for each mouse were collected at the end of experiment and added 0.8 ml ultrapure water containing 6 μg 1,2-^13^C_2_-myristic as an internal standard, vortex for 5 min, followed by centrifugation for 10 min at 13000 g. The supernatants were transferred to a new 1.5 ml tube. 400 μL of supernatants were dried in a SpeedVac sample concentrator and combined with 60 μL methoxyamine hydrochloride in pyridine (10mg/mL), then vortexed for 3 min and shaken at 30 °C for 90 min. 60 μL of BSTFA containing 1% TMCS were added to the sample and shaken at 30 °C for another 60 min. The mixture was then transferred to a sampler vial with a glass insert and subjected to GC-MS analysis. Quality control (QC) samples were prepared by pooling aliquots of all the fecal samples and were processed using same procedure as that for the experimental samples. Analysis was performed on a TRACE 1310 gas chromatograph equipped with an AS 1310 autosampler connected to a TSQ 8000 triple quadrupole mass spectrometer (Thermo Fisher Scientific, Waltham, MA, USA) as described previously 42. Helium was used as the carrier gas, and was maintained at a constant flow of 1.2 mL/min. The oven temperature was initially maintained at 60 °C for 1 min, then increased to 320 °C at 20 °C/min, and then held constant for 5 min. The transfer line temperature between the gas chromatograph and the mass spectrometer was set to 250 °C. Electron impact ionization at 70 eV was employed, with an ion source temperature of 280 °C. Mass spectra were acquired with a scan range of 50-500 m/z and a time range of 3.5-19 min. Raw data were acquired from Xcalibur 2.2 software (Thermo Fisher Scientific) and metabolites were identified through matching of their mass spectra against the reference spectra in the NIST 2014 standard database built-in Xcalibur 2.2 software. Metabolic analyses were performed using MetaboAnalyst 4.0 (https://www.metaboanalyst.ca/). Differential metabolites were identified by fold change >2 and *p-*value <0.05. Pathway enrichment analyses were carried out based on the above differential metabolites.

### Measurement of FITC-Dextran leakage

FITC-Dextran leakage was measured as previously described 43. Briefly, mice were fasted overnight for approximately 6 h and gavaged with FITC-Dextan (Sigma) at a concentration of 50 mg/ml. After 1.5 h, mice were anesthetized using isoflurane and living imaged using IVIS Lumina series Ⅲ (Perkin Elmer) (Exiation: 480 nm, Emission: 520 nm). Serum was collected 4 h after administration of FITC-Dextran and the fluorescence intensity of each sample was measured. A standard curve was performed using FITC-Dextran and the concentration of Dextran in serum was calculated.

### Immunostaining of E-cadherin, Claudin-1 and mucin-2

Colonic tissues were sectioned at 5 μm thickness and underwent antigen retrieval using citrate buffer solution. Tissue sections were then incubated with primary antibodies overnight at 4 °C as follows: anti-E-cadherin (14472, Cell Signaling Technology), anti-Claudin-1 (sc-166338, Santa Cruz) and anti-mucin-2 (sc-7314, Santa Cruz). Alexa Fluor 594 conjugated Donkey anti-Mouse (A-21203, Invitrogen) and Alexa Fluor 488 conjugated Goat anti-Mouse (A-11029, Invitrogen) were used as secondary antibody. Cell images were acquired on a fluorescence microscope (Zeiss, Oberkochen, Germany).

### Alcian Blue staining

Colonic tissues were sectioned at 5 μm thickness, deparaffinized and stained using Alcian Blue staining kit (Leagene, Beijing, China) according to the manufacturer's instructions.

### 16s ribosomal RNA gene sequencing and data analysis

Colon fecal contents were snap frozen with liquid nitrogen and stored at -80 °C. Total genomic DNA was extracted from samples using QIAamp Fast DNA Stool Mini Kit (Qiagen, USA). DNA concentration and purity were monitored on 1% agarose gel. 16S rRNA genes were amplified using specific primer with the barcode. All PCR reactions were carried out using TransStart FastPfu DNA Polymerase (TransGen, Beijing, China). The universal bacterial 16S rRNA gene amplicon PCR primers were used: forward primer was 5'-CCTACGGGNGGCWGCAG-3' and reverse primer was 5'-GACTACHVGGGTATCTAATCC-3'. PCR products were mixed in equidensity ratios. Then, the mixture of PCR products was purified with GeneJET Gel Extraction Kit (Thermo Fisher Scientific, USA). Sequencing libraries were generated using NEB Next Ultra DNA Library Prep Kit for Illumina (NEB, USA) following manufacturer's recommendations and index codes were added. The library was sequenced on an Illumina MiSeq platform. Sequence alignment, operational taxonomic units (OTUs) picking against the SILVA reference collection, clustering, phylogenetic and taxonomic profiling and the analysis of beta diversity were performed with the Quantitative Insights into Microbial Ecology (QIIME) open source software package. Differential genera bacteria were identified using LEfSe analysis and STAMP.

Bacterial metageome content was predicted from 16s rDNA-based microbial compositions, and specific gene abundance were made using PICRUSt2 algorithm. A total of 4905 inferred genes were categorized into 41 KEGG functional pathways. The expressions of purine metabolism-related genes were identified.

### Fecal DNA extraction and quantification of *Lactobacillus spp.* DNA in stools

Total fecal DNA were extracted using E.Z.N.A Stool DNA KIT (Omega Bio-tek, USA). qPCR on a 7500 Sequence Detector (Applied Biosystems, CA, USA) was used to calculate the number of *Lactobacillus spp.* 16S rRNA gene copies in the genomic DNA extracted from stool samples. Samples were quantified in 20 μL reactions using ChamQ SYBR qPCR Master Mix (Low ROX Premixed) (Vazyme, Shanghai, China). Standard curves for quantification consisted of ten-fold serial dilutions in the range of 10^8^-10^1^ copies of the 16S rRNA gene of the stool samples amplified with following primers: Lacto-F (5'-TGGAAACAGGTGCTAATACCG-3') and Lacto-R (5'-CCATTGTGGAAGATTCCC-3'). All measurements were performed in duplicate.

### Measurement of lactic acid concentration in fecals

50 mg colon contents were homogenated with 0.9 ml PBS followed by centrifugation at 5000 g for 15 min. The supernatants were transferred and stored at -80 °C until further experiment. Lactic acid concentration was assessed using Lactic acid Detection Kit (SenBeiJia Biological Technology Co., Ltd., Nanjing, China) according to the manufacturer's instructions and normalized by the weight of colon contents.

### Uric acid detection in cultured cells

*Lactobacillus sp.* were purchased from BeNa Culture Collection (Beijing, China), maintained under anaerobic condition and grown in MRS media as previously reported. MRS media containing 2 μM, 4 μM and 8 μM Rhein were used and maintained for 24 h. The supernatants were collected, centrifuged at 13000 g for 10 min and stored at -20 °C. To detect uric acid levels in human NCM-460 cells, *Lactobacillus sp.* were cultured. Once the OD_600nm_ reached 2.0, Lacto were centrifuged at 13000 g for 10 min to remove bacteria and the supernatants were collected, stored at -20 °C and used within 3 days. 5×10^5^ NCM-460 cells were either treated with 8 μM and 16 μM rhein or Lacto condition media 50 μL for 24 h. After 24 h, the supernatant was collected and uric acid concentration was detected using QuantiChrom Uric Acid Assay Kit (BioAssay Systems).

### qPCR analysis

To quantify the *XDH* gene expression, 1×10^6^ NCM-460 cells were treated with 8 μM and 16 μM rhein for 24 h. Total RNA was extracted using RNAiso Plus reagent (Takara, Japan) according to the manufacturer's instructions. cDNA was synthesized from 500 ng total RNA using Hiscript®II QRT SuperMix (Vazyme, Shanghai, China). Real-time PCR was performed using ChamQ SYBR qPCR Master Mix (Low ROX Premixed) (Vazyme, Shanghai, China) and detected by ABI 7500 system (Applied Biosystems, CA, USA). Primers were used as follows:

- *XDH*-F: (5'-AGCTCTGAAAATCCCCACCTC-3');
- *XDH*-R (5'- CAAGATGGTCTGACAAGCCG-3');
- GAPDH-F: (5'-ACAACTTTGGTATCGTGGAAGG-3');
- GAPDH-R: (5'-GCCATCACGCCACAGTTTC-3').

Gene expression was normalized by GAPDH.

### Fecal microbiota transplantation

Fecal transplantation was performed based on an established protocol 44. Briefly, stools from rhein treated mice or DSS mice were collected, snap frozen and stored at -80 °C. The stools from donor mice of each group were pooled and 100 mg was resuspended in 1 ml of sterile saline. The solution was vigorously mixed for 10 s and centrifuged at 800×g for 3 min. The supernatant was collected and used as transplant material. Fresh transplant material was prepared on the same day of transplantation within 10 min before oral gavage to prevent changes in bacterial composition.

Mice were fed with antibiotic water (1g/L ampicillin, 1g/L metronidazole, 0.5 g/L vancomycin and 0.5 g/L neomycin) for 5 days. Transplantation was performed by oral gavage of 200 μL transplant material during chronic colitis once per week.

### Statistical analysis

All data are presented as mean ± standard deviation (SD). The data were analyzed using two-tailed Student's *t*-test between two groups and one-way analysis of variance followed by Dunnett's post hoc tests when groups were more than two. *P* < 0.05 was considered statistically significant.

## Supplementary Material

Supplementary figures.

## Abbreviations

IBD: inflammatory bowel disease
DSS: dextran sulfate sodium
Th17: T helper 17
16s rDNA: 16s ribosomal DNA
UC: ulcerative colitis
NF-kappaB: nuclear factor kappa-B
MAPK: mitogen-activated protein kinase
Nrf2: Nuclear factor erythroid2-related factor 2
IKKβ: inhibitor of nuclear factor kappa-B kinase
LCN2: lipocalin 2
IL-17: interleukin 17
MLN: mesenteric lymph nodes
PCA: principle component analysis
XDH: xanthine dehydrogenase/oxidase
LefSe: linear discriminant analysis effect size
LDA: linear discriminant analysis
STAMP: sequence tag-based analysis of microbial populations
FMT: fecal microbiota transplantation
PICRUSt2: Phylogenetic Investigation of Communities by Reconstruction of Unobserved States 2
