# Supplementary material for: Rhein modulates host purine metabolism in intestine through gut microbiota and ameliorates experimental colitis
Source: Theranostics. 2020 Aug 29;10(23):10665–79. doi: 10.7150/thno.43528 (PMC7482825; doi:10.7150/thno.43528)
Supplement: Supplementary file 1 — Supplementary figures. [file thnov10p10665s1.pdf]

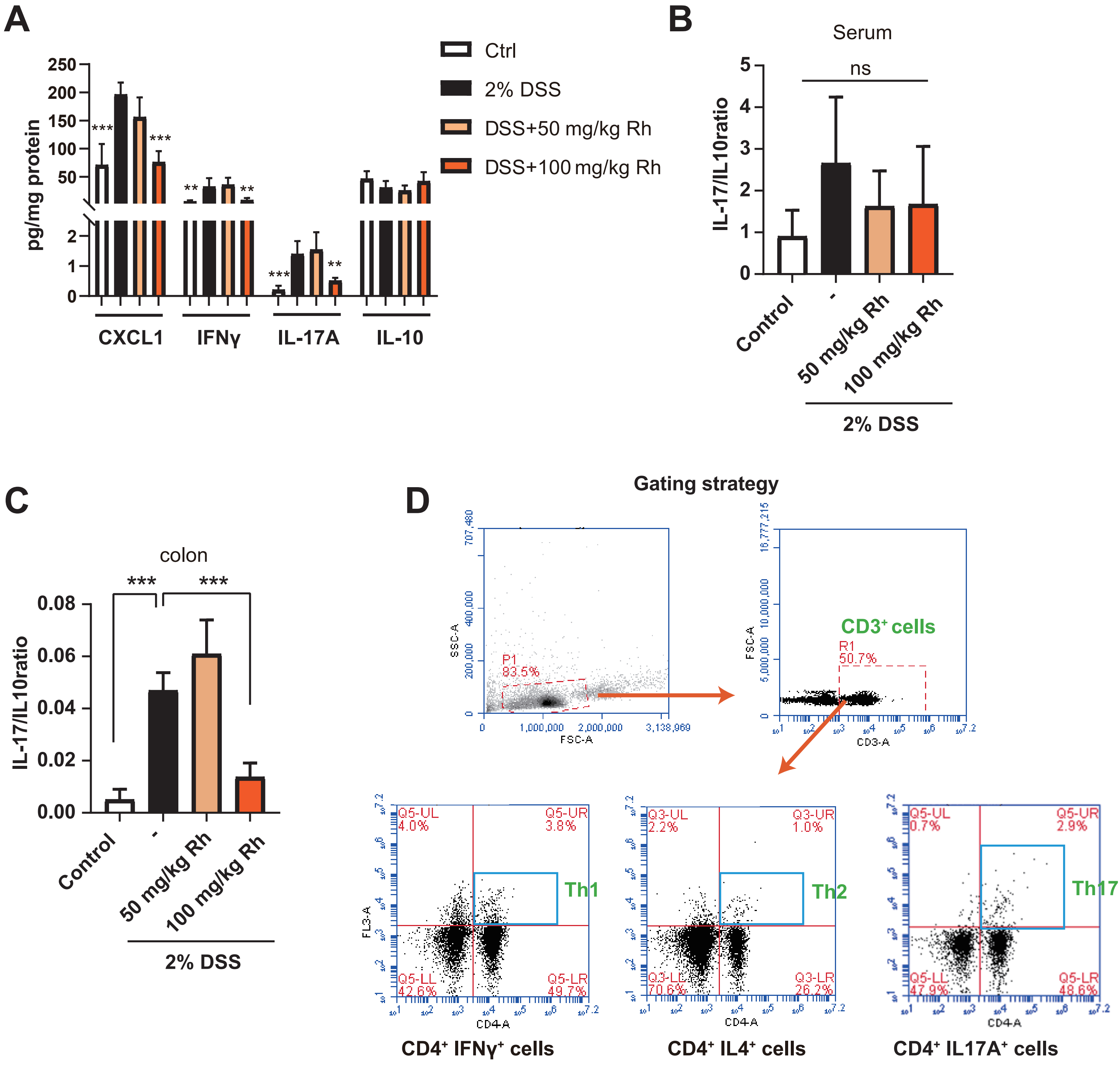

Figure S1. Rhein exhibited anti-inflammatory effects against DSS-induced colitis. (A) CXCL1, IFNγ, IL-17A and IL-10 concentrations in colon tissues. (B) Serum ratio of IL-17/IL-10. (C) Ratio of IL-17/IL-10 in colon tissues. (D) Gating strategy. $CD3^+$ cells were used for further gating. Th1: $CD4^+IFN\gamma^+$, Th2: $CD4^+IL\text{-}4^+$, Th17: $CD4^+IL\text{-}17A^+$.*P<0.05, **p<0.01, ***p<0.001 versus DSS. At least two independent experiments were performed.

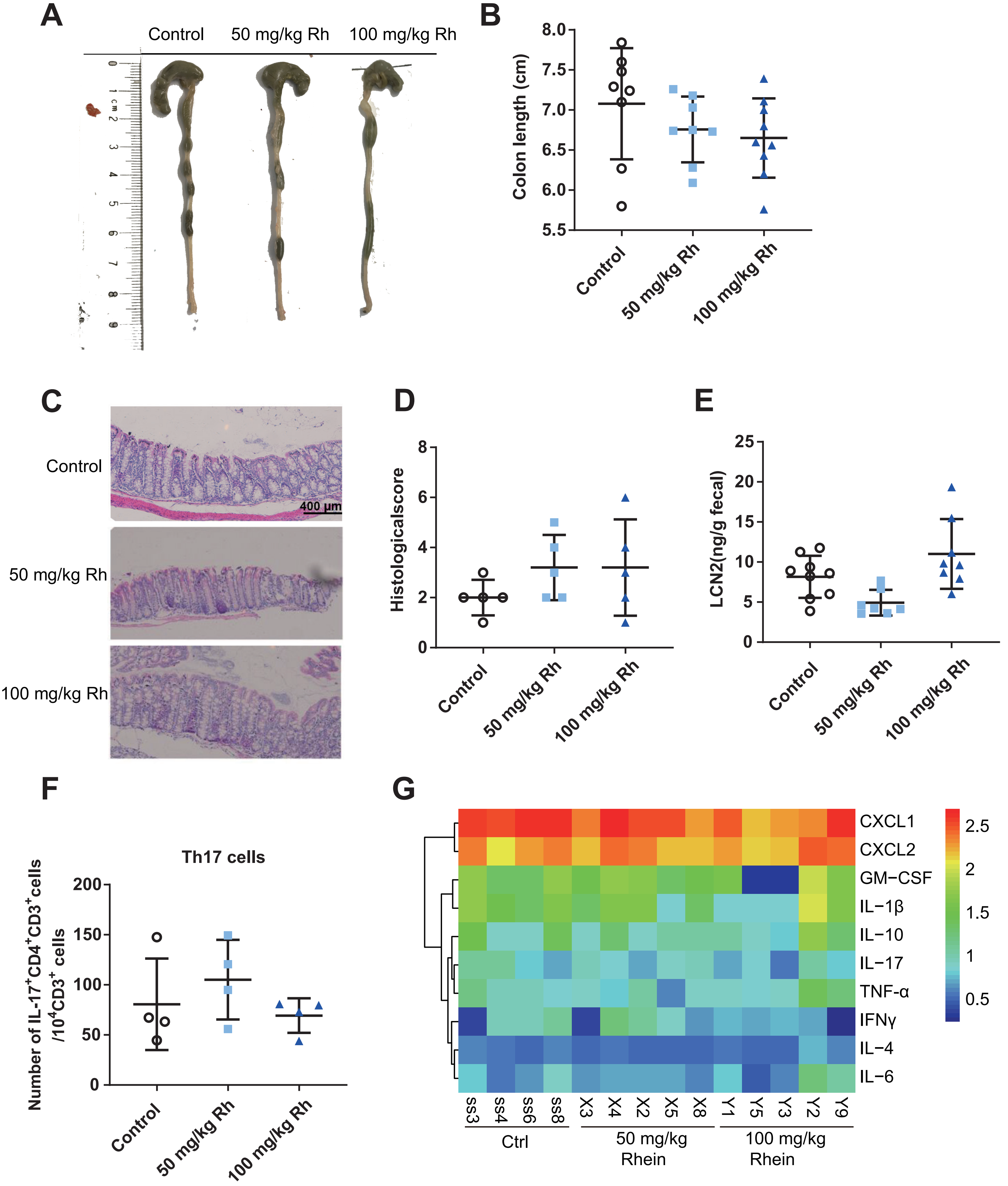

Figure S2. Rhein treatment alone did not cause a noticeable inflammatory response. (A) Representative colon pictures from each group. (B) Colon length in each group (n = 8). (C) Representative H&E staining of colon tissue sections from each group. Scale bar, 400 μm. (D) Histological score in each group (n = 5). (E) Fecal level of LCN2 in each group (n = 7-9). (F) Heatmap of ten specific cytokines levels in serum, including KC, MIP-2, IFN-γ, IL-4, IL-6, GM-CSF, IL-1β, IL-17, IL-10, and TNF-α (n = 4-5). (G) Flow cytometry analysis of Th17 cells per 10^4 $CD3^+$ cells in each group (n = 4). At least two independent experiments were performed.

A

Key metabolites (differed by Fold change and P-value)	DSS+100 mg/kg Rhein/DSS
Up-regulated	glutamic acid, orotic acid, taurine, 3-4-dihydroxybenzoic acid, arabitol, aspartate, 3-hydroxy-3-methylglutaric acid, 5-aminovaleric acid, hypoxanthine, xanthosine, alpha-ketoglutarate, galacturonic acid, azelaic acid
Down-regulated	Inosine, 5-alpha-cholestan-3-beta-ol, 2-ketoisocaproic acid, 2'-deoxyguanosine, cholesterol, alpha tocopherol, linoleic acid, beta-hydroxybutyric acid, 2-butyne-1-4-diol, m-cresol

B

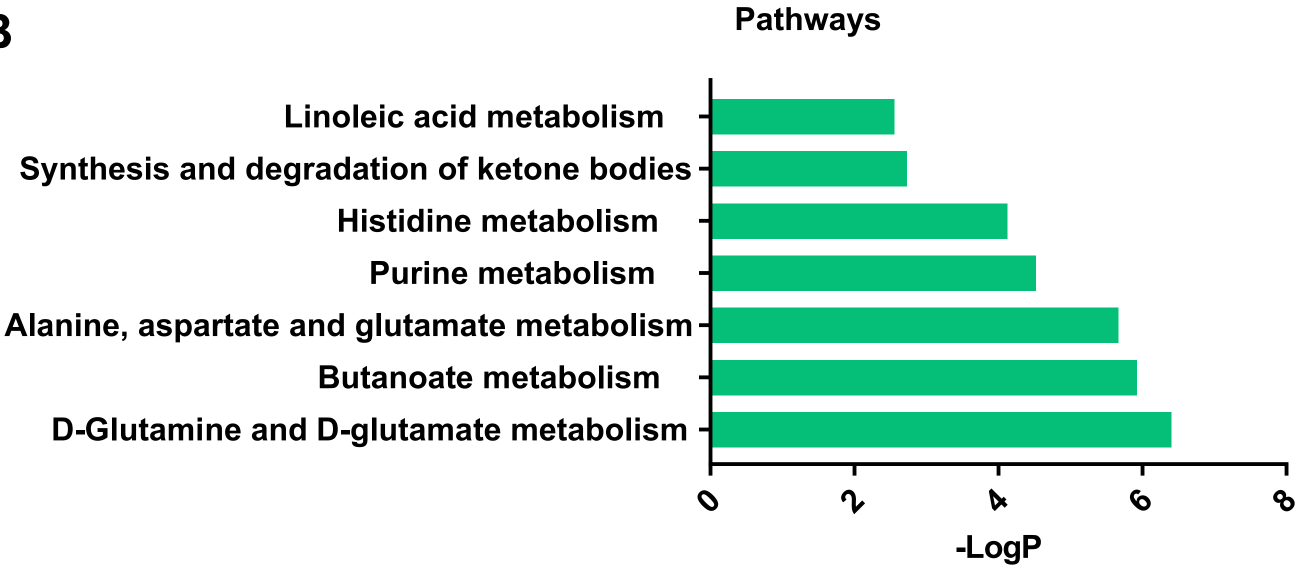

Figure S3. Key metabolites and pathway enrichment analysis based on metabolomics. (A) Key metabolites between rhein and DSS groups, selected by fold change>2 and p value<0.05. (B) Enriched pathways based on different metabolites in (A) using MetaboAnalyst 4.0. Pathways with a p-value<0.05 are shown.

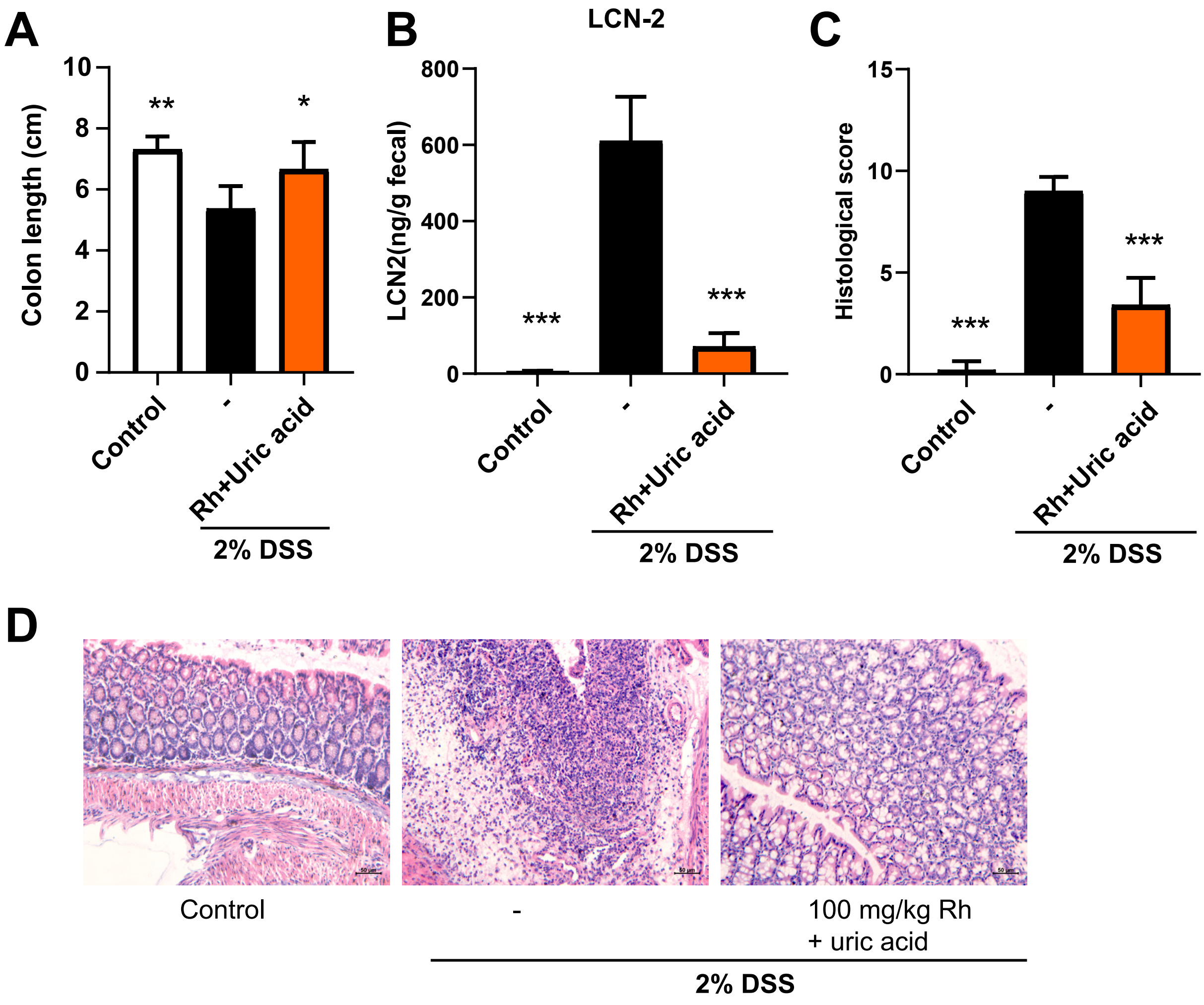

Figure S4. Uric acid treatment did not affect the protective effects of rhein. 100mg/kg rhein and 1g/kg uric acid were orally gavaged daily after colitis induction. (A) Colon length in each group (n = 5). (B) Fecal level of LCN2 in each group (n = 5). (C) Histological score in each group (n = 4). (D) Representative H&E staining of colon tissue sections from each group. *p<0.05, **p<0.01, ***p<0.001 versus DSS group. At least two independent experiments were performed.

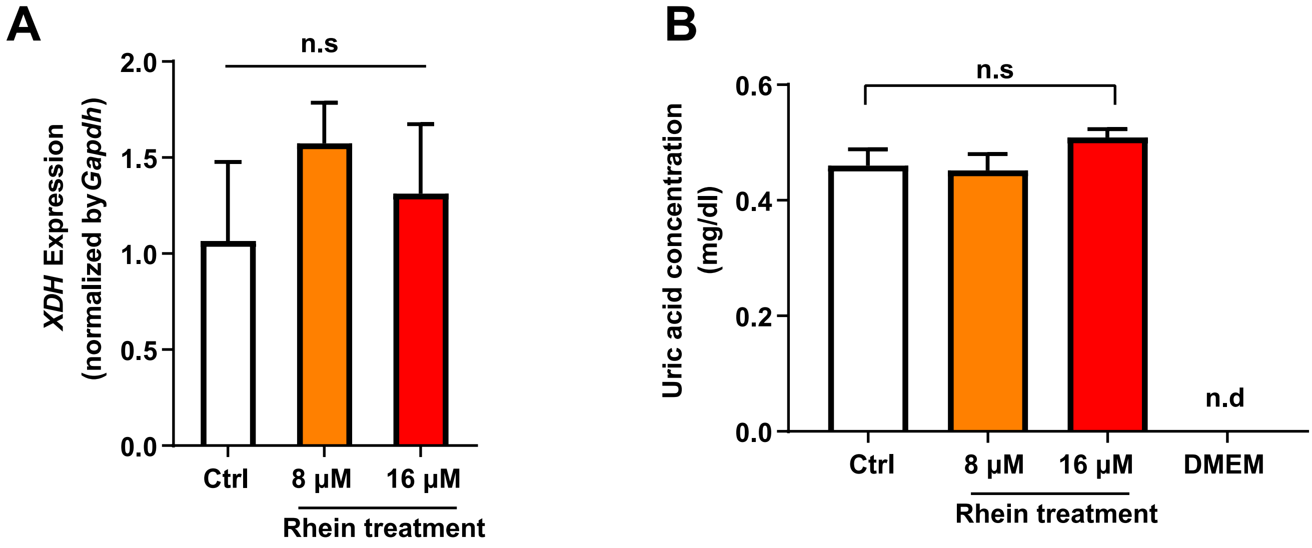

Figure S5. Rhein had no direct effects on uric acid production. (A) NCM-460 cells were treated with 8 μM or 16 μM rhein and *XDH* gene expression was determined by qPCR (n = 3). (B) NCM-460 cells treated with 8 μM or 16 μM rhein for 24 h and uric acid concentration was determined (n = 3). Ctrl: control; *XDH*: xanthine oxidoreductase n.s: not significant; n.d: not detected. Three independent experiments were performed.

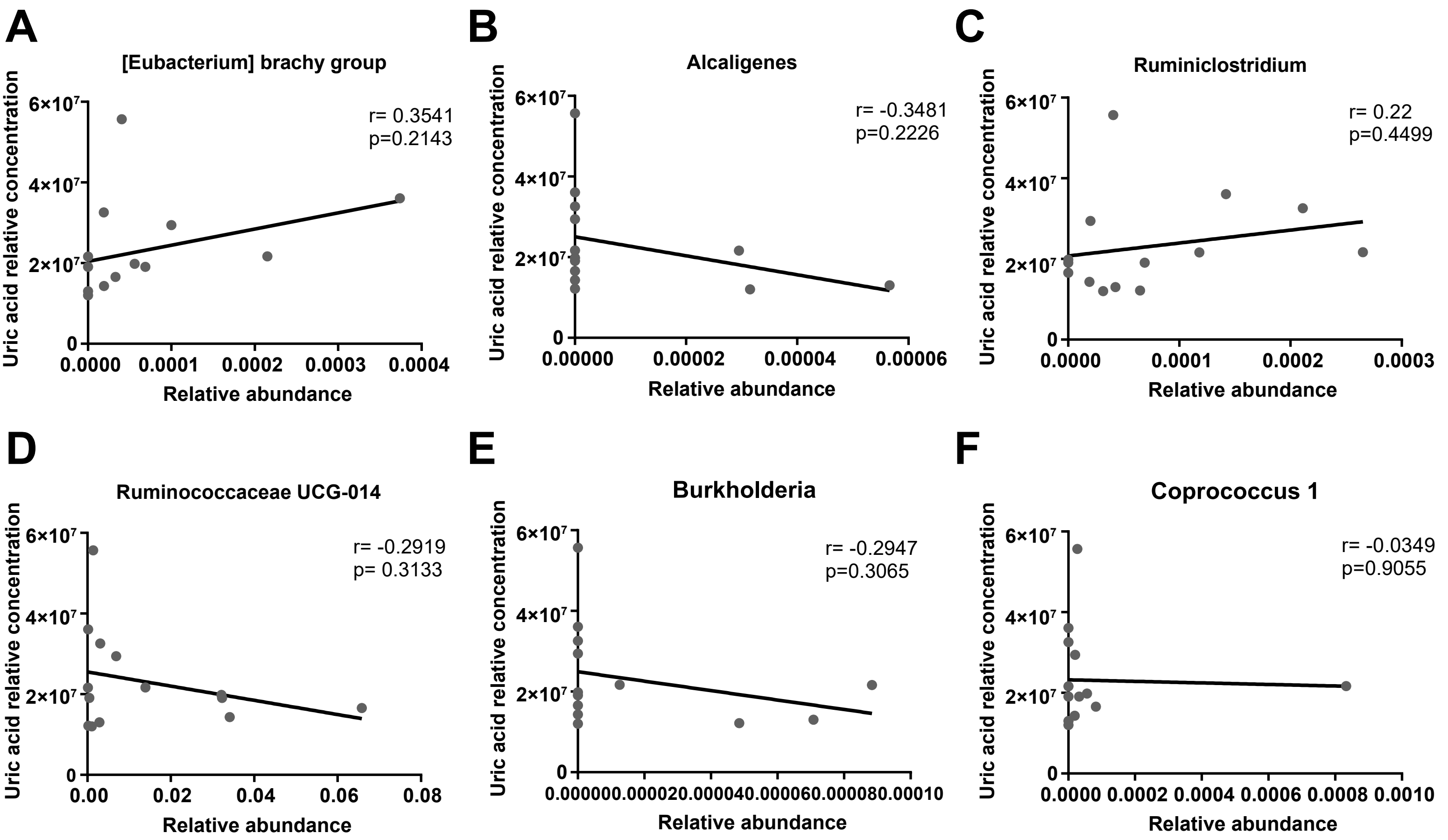

Figure S6. Spearman's correlation analysis. Different bacteria abundance identified by LEfSe analysis and paired uric acid levels. P value<0.05 was considered significant.

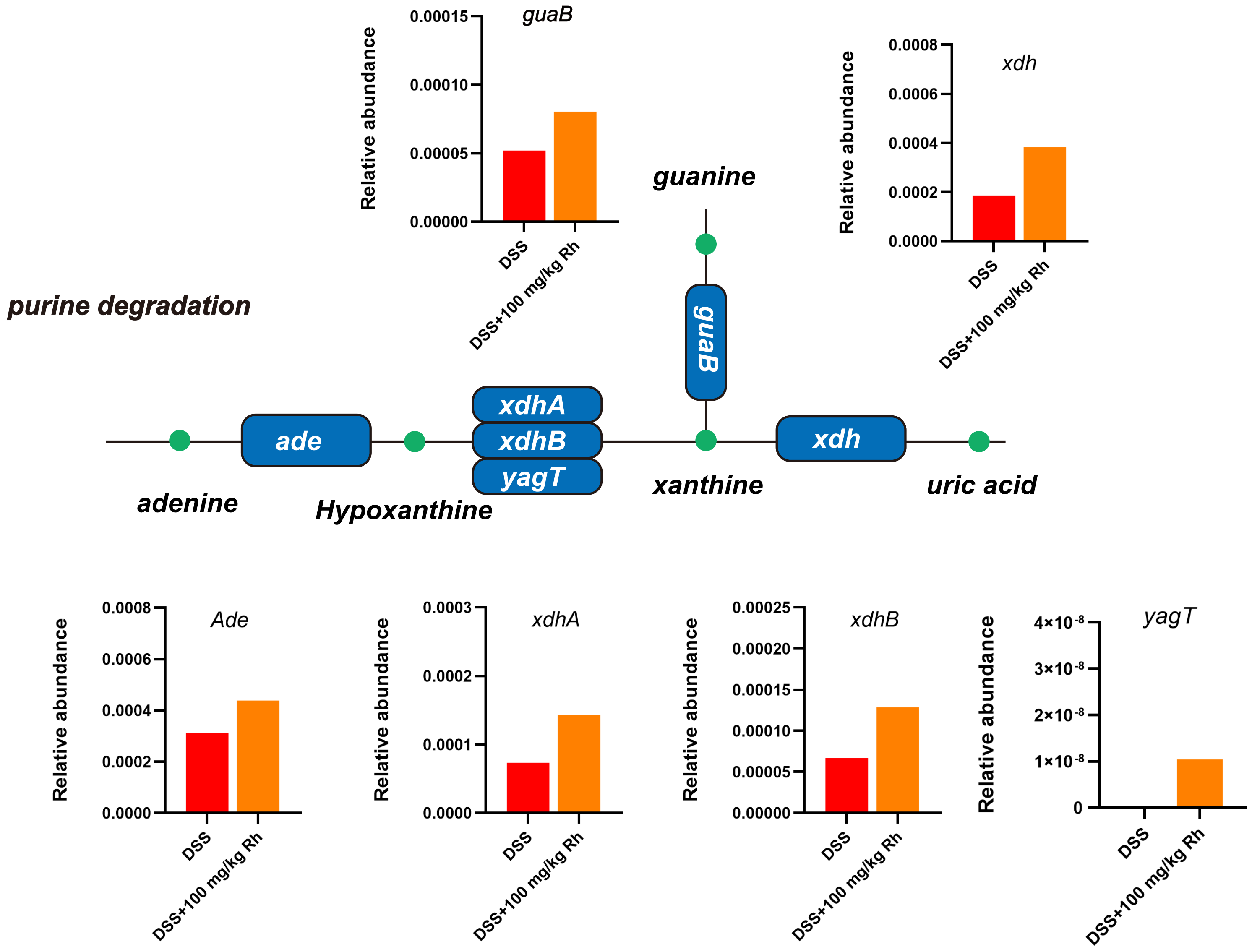

Figure S7. Microbial purine metabolism pathway analysis. PICRUSt2 was used. Relative abundance of genes involved in purine metabolism and representative process of microbial purine degradation are shown. Each box represents a gene. No significance was observed.

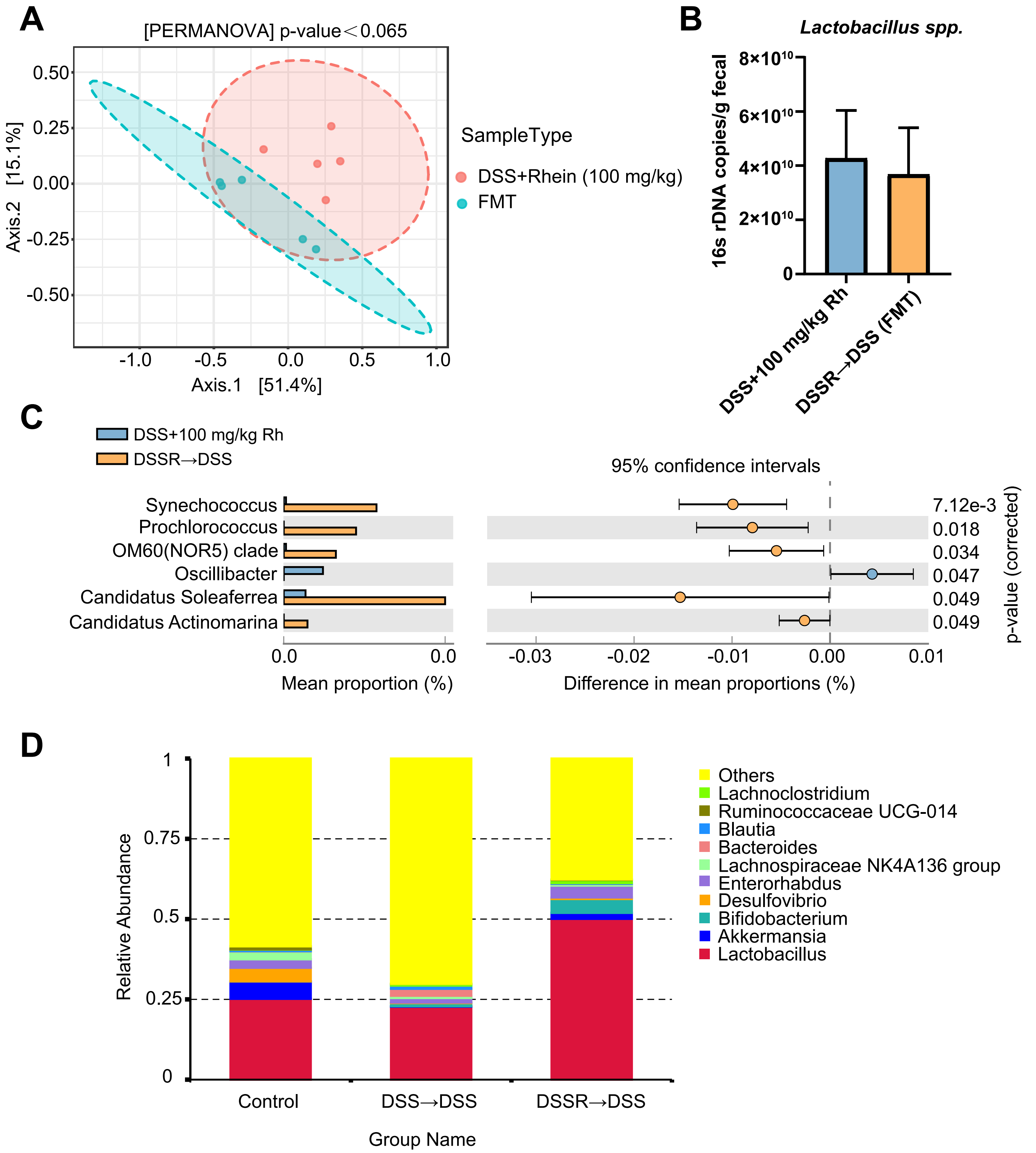

Figure S8. FMT revealed the similar microbiota profile to rhein treatment. (A) UniFrac-based PCoA analysis with the PERMANOVA significant test of each group (n = 5). (B) 16s rDNA copies of *Lactobacillus spp.* measured by quantitative PCR. (C) STAMP analysis uncovered the differences between DSS+Rhein and FMT (DSSR→DSS) groups. Genera with a significance of P<0.05 are shown. (D) Relative abundance of microbial taxa was determined on genus level. Top 10 abundant genus were shown.
